# Stable hovering of a jellyfish-like flying machine

**DOI:** 10.1098/rsif.2013.0992

**Published:** 2014-03-06

**Authors:** Leif Ristroph, Stephen Childress

**Affiliations:** Applied Math Lab, Courant Institute, New York University, 251 Mercer St., New York, NY 10012, USA

**Keywords:** micro air vehicle, flight stability, flight control, biomimetics, unsteady aerodynamics

## Abstract

Ornithopters, or flapping-wing aircraft, offer an alternative to helicopters in achieving manoeuvrability at small scales, although stabilizing such aerial vehicles remains a key challenge. Here, we present a hovering machine that achieves self-righting flight using flapping wings alone, without relying on additional aerodynamic surfaces and without feedback control. We design, construct and test-fly a prototype that opens and closes four wings, resembling the motions of swimming jellyfish more so than any insect or bird. Measurements of lift show the benefits of wing flexing and the importance of selecting a wing size appropriate to the motor. Furthermore, we use high-speed video and motion tracking to show that the body orientation is stable during ascending, forward and hovering flight modes. Our experimental measurements are used to inform an aerodynamic model of stability that reveals the importance of centre-of-mass location and the coupling of body translation and rotation. These results show the promise of flapping-flight strategies beyond those that directly mimic the wing motions of flying animals.

## 1. Introduction

In our quest to build miniature and manoeuvrable flying machines, it is natural to look to insects as a source of inspiration [1]. Driven by this goal to reverse-engineer Nature's flyers, the last two decades have seen rapid progress in understanding the aerodynamics of flapping wings [2–4] as well as the behavioural aspects of insect flight [5–8]. In some ways, we have reached a stage similar to that encountered by the Wright brothers in their efforts to achieve aeroplane flight. The Wrights focused on control and stability, eventually implementing the strategy of wing warping that was inspired by observations of soaring birds [9]. Stabilization of flapping-wing aircraft presents unique challenges, including unsteady aerodynamics, small length scales and fast time scales. Addressing these issues is likely to require exploration of many approaches, ranging from mimicking insects to inventing new flight schemes.

Previous and ongoing efforts to construct hovering ornithopters, or flapping-wing aircraft, have taken the biomimetic approach that aims to imitate the wing motions of insects. Most designs are based on the so-called normal hovering [10–18], the mode employed by flies, bees, moths and hummingbirds [1–4]. Wings are flapped back and forth in a horizontal stroke plane and rapidly flipped over at each stroke reversal. The aerodynamics of these motions has been clarified by scaled experiments and flow simulations, including studies that have revealed an intrinsic instability in body orientation [19–21]. Hence, to keep upright, these insects require sensory–motor systems that provide active modulation of flight forces [5,8]. Normal hovering robots also exhibit this instability and tend to flip over if left uncontrolled [12,13,16,17]. Stabilizing these designs has demanded either feedback control systems [10,15,18] or the addition of tails or sail-like surfaces that act as aerodynamic dampers [13,14,16,17]. The second mode of hovering is represented by the up-and-down flapping of the dragonfly, in which the broadside of each wing is presented during the downstroke followed by a slicing motion upwards [22]. Less is understood about the stability of this mode, though both dragonflies [23] and robotic designs [24] appear to rely on active control.

Here, we aim to achieve stable hovering using flapping wings alone, without the need for feedback control and without aerodynamic dampers. Such a minimalistic design could prove particularly useful as robots are further scaled down, in which case implementing control systems would be increasingly challenging and damping surfaces would undermine both miniaturization and manoeuvrability. To this end, we design and construct a mechanical flyer that employs a new mode of hovering, with wing motions that are not used by insects or birds. Force measurement and high-speed video allow us to characterize the ornithopter's lift generation and free-flight stability properties. Finally, these experimental measurements are used to inform a mathematical model that reveals the aerodynamic basis of stability.

## 2. Concept, construction and wing kinematics

**Figure 1.**
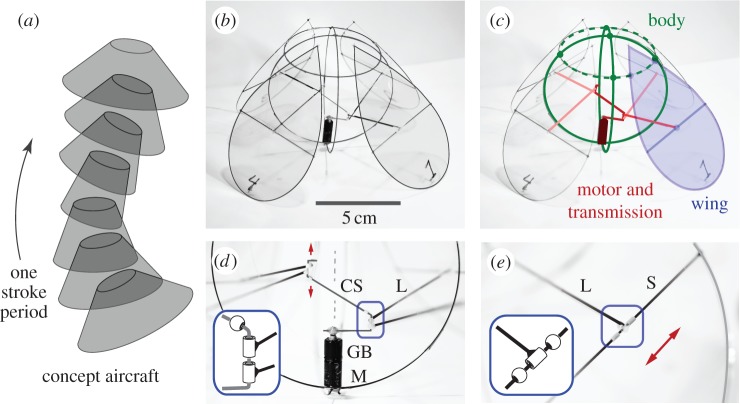
A flying machine. (*a*) A concept flyer opens and closes a surface. (*b*) The prototype uses a motor to pull in and push out four wings. (*c*) The carbon fibre body (green) consists of two crossed vertical loops that support the motor (dark red) below and a horizontal upper loop (dashed). Each wing (blue) is a Mylar-covered frame that is hinged on this upper loop. (*d*) A motor (M) with gearbox (GB) rotates a crankshaft (CS), which connects via a link (L) to each wing. Flapping amplitudes can be adjusted by bending up or down (arrows) the upper arm of the crankshaft. (*e*) The link position along the wing spar (S) can be adjusted (arrow), modifying the chordwise motion of the wing. Insets: rotary joints are made from segments of Teflon tubing.

Our approach is motivated by experiments conducted in our Applied Math Lab at New York University in which cone- and pyramid-shaped bodies are observed to hover within a vertically oscillating airflow [25,26]. These passive flyers rely on the externally imposed flow to generate lift as well as the self-righting aerodynamic torque needed to keep upright [27]. Our concept vehicle is an active analogue that would achieve stable hovering by flapping an aerodynamic surface. In figure 1*a*, we illustrate this concept as a conical surface that reciprocally opens and closes. While there is no known flying animal that employs such a scheme, this design is reminiscent of the swimming motions of jellyfish [28,29].

To realize this concept, we have constructed an ornithopter that uses a motor to drive inward-and-outward oscillations of four wings. As shown in the image in figure 1*b* and the schematic in figure 1*c*, the motor sits low on the body, which consists of two crossed vertical loops of carbon fibre. These loops support an upper horizontal loop that serves as a fulcrum for the wings. Each wing itself is also a loop of fibre spanned by thin Mylar film, hinged near its top and connected to the crankshaft via a link to a spar. As the motor rotates, each wing is pulled in and pushed out. All rotary joints are made of short segments of low-friction Teflon tubing, as shown in figure 1*d,e*. Also, this simple drive mechanism does not close all four wings simultaneously but rather causes one opposing pair to lead the other by a quarter period. To achieve the low body mass of 2.1 g, we have used lightweight construction materials as well as a 1.1 g motor pre-assembled with a gearbox. Finally, we have not yet fitted the prototype with a battery and instead used an external power supply wired to the motor, allowing us to explore how the wing motions and lift vary with the driving voltage and flapping frequency.

**Figure 2.**
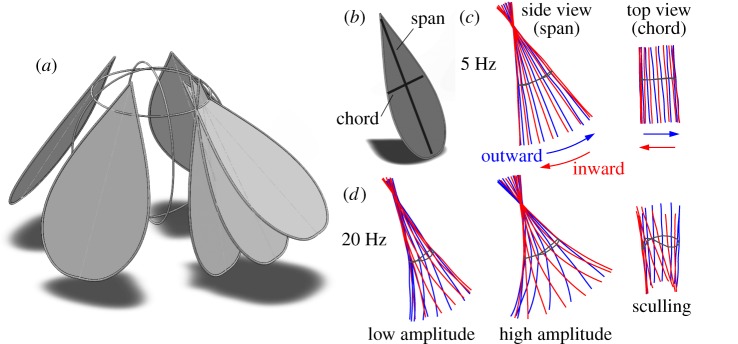
Wing flapping motions. (*a*) Schematic showing the inward-and-outward flapping motion. For clarity, only one wing is shown at its inward, mid-stroke and outward orientations in the flapping cycle. (*b*) Span and chord axes. (*c*) Flapping motions at low frequency, as measured from high-speed video. The black line indicates the trajectory of the link–spar connection point at which the wing is driven. (*d*) At higher frequency, the wings bend along their span, and the flapping amplitude can be adjusted as described in figure 1*d*. The chordwise motion can be adjusted as per figure 1*e*: the driving point is offset from the centre, yielding sculling motions that generate chordwise force. (Online version in colour.)

We display a schematic of the inward–outward flapping motions in figure 2*a*, and the actual wing motions are extracted from high-speed video of the ornithopter powered at varying voltages (see the movies in the electronic supplementary material). At low voltage and thus low flapping frequency of 5 Hz (figure 2*c*), the wings remain rigid as they reciprocally oscillate in and out. Here, the dark trajectory represents the link–spar connection point at which the wing is driven. At higher voltage and thus higher frequency (figure 2*d*), the span of the wing flexes strongly during both half-strokes. Furthermore, we have developed mechanisms to adjust the flapping amplitude and chordwise motions of the wings. These adjustments are critical to inducing manoeuvring modes and achieving the equilibrium needed to hover, two issues that are discussed in detail in later sections.

## 3. Motor characteristics, wing size and lift

**Figure 3.**
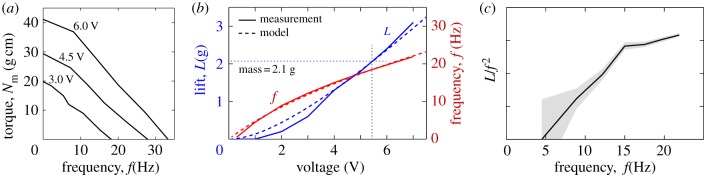
Lift generation. (*a*) Torque–frequency curves of the motor for several values of the applied voltage. (*b*) Lift (*L*) and flapping frequency (*f*) measured for the flyer inverted on a weighing scale. At 5.5 V, the flyer generates lift equal to body weight. An aerodynamic model is fitted to the hovering data and predicts the dependence of frequency and lift on voltage (dashed lines). (*c*) The lift coefficient increases with frequency, an enhancement likely to be due to wing bending. The grey area reflects the ±0.05 g error in force measurement. (Online version in colour.)

In determining the size and lifting capacity of our prototype—which has a wing length of 8 cm—we were guided by trial and error as well as scaling considerations. Experimentally, we found that there has to be a wing size that seems well suited to the motor, with both smaller and larger wings generating weak lift. Intuitively, small wings can be flapped at high frequency but suffer from small area, while large wings can only be flapped slowly by the power-limited motor. This trade-off can be characterized by the torque–frequency curve of the motor (Solarbotics, GM15), which we measured for several voltages, as displayed in figure 3*a*. For a given voltage, the motor delivers the highest power when running near the middle of its speed range [30]. Near 6 V, for example, this corresponds to our motor spinning at frequency *f*_m_ ∼ 15 Hz and delivering a torque of *N*_m_ ∼ 25 g cm. Some particular value of the wing size *R* will lead to flapping at *f*_m_, with the motor torque balancing the aerodynamic torque, 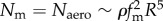 [31]. Here, *ρ* is the density of air, and the torque is derived from the scaling of aerodynamic forces at high Reynolds number (*Re* = *ρfR*^2^/*μ* ∼ 10^4^, where *μ* is the viscosity of air). Thus, the ideal wing length is predicted to be of the order of 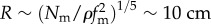, which is comparable to the value determined by trial and error.

This analysis also predicts a lift 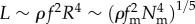 of several grams, suggesting that this motor is indeed capable of supporting body weight. We tested this by inverting the ornithopter on a scale to measure the average force generated, and figure 3*b* shows the increasing lift (*L*) and flapping frequency (*f*) for increasing voltage. Critically, a voltage of 5.5 V leads to a frequency of 19 Hz and a force of 2.1 g, which is sufficient to balance weight during hovering. These measurements and scaling analysis suggest that the size and lift of a hovering machine is strongly dependent on its motor properties, and these ideas are incorporated into an aerodynamic model described below. Furthermore, we note that the scaling of lift as *L* ∼ *f*^2^ strictly applies to wings of fixed shape, and departures from this behaviour can be used to assess the effect of wing flexibility. As shown in figure 3*c*, the ratio *L*/*f*^2^ increases with frequency for our flyer, suggesting that the wing bending shown in figure 2*d* leads to a lift enhancement [32,33].

## 4. Manipulation of wing motions to achieve manoeuvring modes

Having achieved the required lift, we next trimmed the aircraft, that is, ensured equilibrium of spin and tilt torques [34]. The importance of trimming became clear as our first prototype that generated strong lift nonetheless rapidly spun and tumbled over when released. If the ornithopter tended to tilt one way, we compensated by increasing the flapping amplitude of the wings on this side, with example wing motions shown in figure 2*d*. This adjustment was accomplished by bending up or down the upper arm of the crankshaft prior to a test flight, as shown in figure 1*d*. Similarly, if the ornithopter tended to spin, we adjusted the chordwise motion to generate a compensating torque. Specifically, the sculling motions shown in figure 2*d* are induced by sliding the link–spar connection point along the chord of the wing (figure 1*e*).

**Figure 4.**
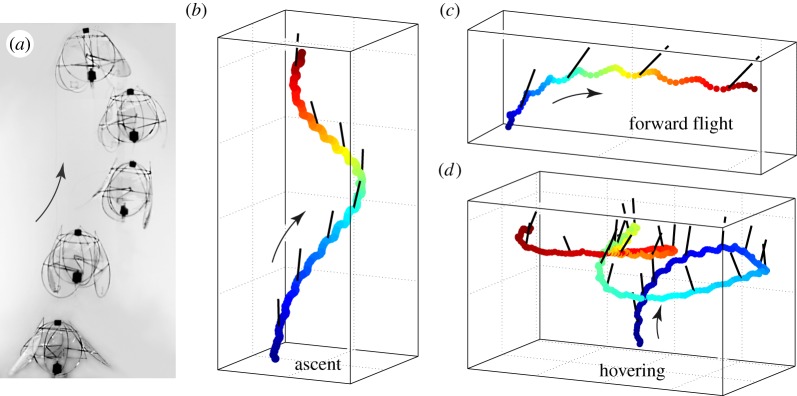
Free-flight trajectories measured from high-speed video. (*a*) Snapshots of every four wing-beats during ascending flight. Black markers are automatically tracked in two views to determine the body centre-of-mass position and tilt. (*b*) Three-dimensional reconstruction of a spiralling ascent, with position shown coloured in time (blue to red) and tilt shown as a black line every other wing-beat. Grid lines are 10 cm apart. (*c*) Transition to forward flight with tilt towards the direction of motion. (*d*) Hovering flight consists of erratic runs and loops.

We then test-flew the trimmed ornithopter in an arena that allowed us to measure its free-flight dynamics. Markers were added to the body, two views were captured on high-speed video, and a custom code tracked the markers and determined the body centre-of-mass position and tilt orientation. We took advantage of the ornithopter's adjustability to access manoeuvers; for example, attaining ascending flight by applying a high voltage (see the movie in the electronic supplementary material). In figure 4*a*, we show snapshots taken of the climbing flight, and in figure 4*b* we plot the reconstructed flight path. The centre-of-mass trajectory is colour-coded in time from blue to red, and the tilt orientation every other wing-beat is shown as a black line. Small oscillations represent movements within a wing-beat, and the helical or spiral-like trajectory is likely to be the result of imperfect trimming. To access the second flight mode, we increased the flapping amplitude of the wings on one side of the body, causing the craft to tilt over and fly in a directed path in the horizontal plane. Figure 4*c* shows the flyer's trajectory as it transitions to steady forward flight. These modes show the potential to implement navigation and control schemes in future versions of the ornithopter. Most importantly, these data show that the flyer has an inherent tendency to keep upright during manoeuvers.

## 5. Stability of hovering

As the final demonstration of its capabilities, we sought hovering flight by trimming spin and tilt and setting the voltage to just over 5.5 V. Once powered, the ornithopter rose upwards for several wing-beats and then maintained a relatively constant height while meandering in the horizontal plane, as shown in figure 4*d* and the movie in the electronic supplementary material. The flight path is marked by sequences in which the flyer tilts to one side and translates in that direction before returning to a near upright posture. The succession of these runs and loops leads to an erratic path reminiscent of the fluttering flight of a moth.

**Figure 5.**
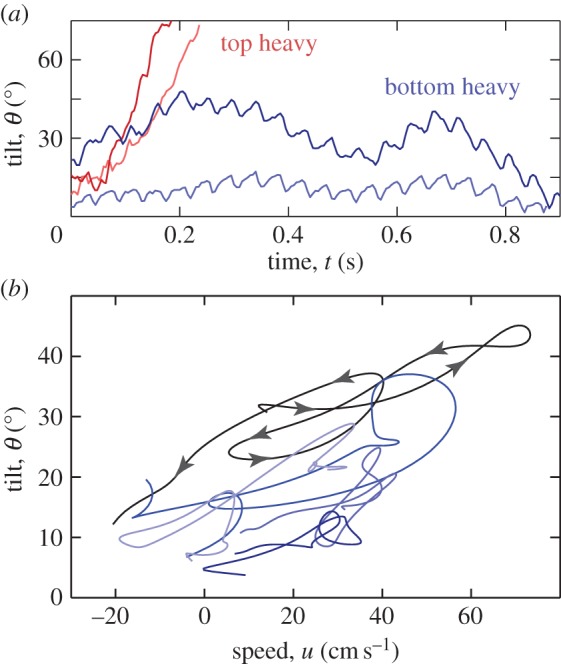
Stability during hovering. (*a*) Typical tilt dynamics measured from high-speed video. The flyer has a low centre of mass (bottom heavy) and undergoes stable oscillations. An earlier prototype with a motor attached high on the body (top heavy) flips over in a few wing-beats. (*b*) Tilt is correlated with horizontal speed for five hovering sequences. Excursions to high tilt are accompanied by high speed, causing the flyer to return to low angle. (Online version in colour.)

The flyer recovers from excursions to large tilts, as shown by the example dynamics shown in figure 5*a* (bottom heavy). Here, the high-frequency fluctuations represent the motion within a wing-beat, while the slower undulations correspond to the tilt–run–recover sequences. We quantify these observations in figure 5*b*, which reveals that the tilt angle is correlated with horizontal speed for five cases of hovering. In any given sequence—such as the dark curve marked with arrows—the flyer tilts over while gaining speed and then returns to lower angles as it slows down. The second clue to the stabilization mechanism comes from an early prototype designed with its motor fixed at the top of the body frame. This top-heavy version rapidly tumbles over when released, as revealed by the example dynamics shown in figure 5*a* (top heavy). These observations indicate the importance of the centre-of-mass location and the coupling of body degrees of freedom, and these ideas are incorporated into the stability model below.

## 6. Aerodynamic model of flight forces

**Figure 6.**
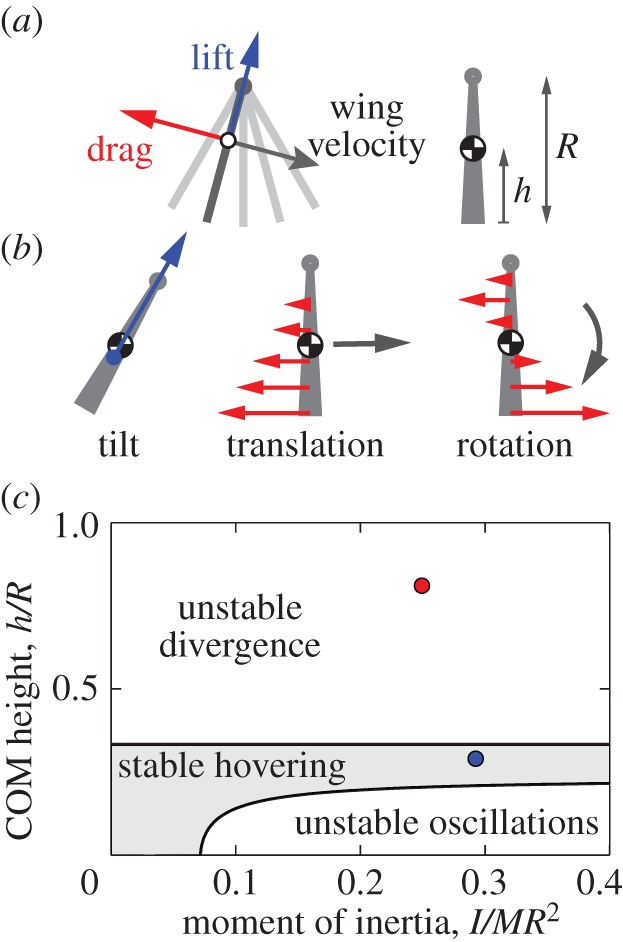
Model of aerodynamic forces and flight stability. (*a*) For each point along a hinged flapping wing, the aerodynamic force is decomposed into lift and drag. The model presented in the electronic supplementary material computes the average forces for small-amplitude flapping, and stability is shown to depend on the centre-of-mass (COM) height relative to wing length, *h/R*, as well as moment of inertia, *I*. (*b*) Wing forces are modified as a result of tilt, translation and rotation. A tilt causes acceleration to the side, sideways motion induces a resistive drag along each wing and body rotations also alter the drag. (*c*) Stability diagram of hovering. The nominal bottom-heavy prototype (blue dot) is predicted to be stable, and the top-heavy version (red) is unstable.

To understand the lift and stability properties of our flyer, we formulated an aerodynamic model for the forces on the flapping wings. Here we outline the central ideas of the model, with supporting calculations presented in full in the electronic supplementary material. We first note that a wing undergoing steady motion experiences a force that is proportional to the product of its area and the square of its speed [31]. For a rigid wing hinged at its top and driven to flap back and forth, as shown in figure 6*a*, the force is expected to follow similar scaling. This motivates a formalization in which the fluid force on a given segment along the span is also proportional to the product of area and square of the instantaneous speed, and indeed such quasi-steady aerodynamic models have been used to analyse insect flight [7,8,20,21]. Lift is defined to be the component of force pointing perpendicular to the wing velocity ***ν***, and its magnitude on a blade element of area d*S* is 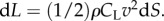 Similarly, *drag* is the component antiparallel to velocity, and it has magnitude 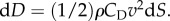 Here, *C*_L_ and *C*_D_ are lift and drag coefficients, respectively, and integrating these expressions along the wings provides estimates for the flight forces in terms of these parameters.

Our measurements of motor torque, flapping frequency and lift shown in figure 3*a,b* allow us to uniquely determine the values of these force coefficients. In particular, the motor must overcome wing drag as well as the inertial resistance associated with accelerating the wing mass and the added mass of the surrounding air. Equating the motor torque with the time-averaged aero-inertial torque yields a prediction for the flapping frequency as a function of voltage, with *C*_D_ as a parameter (see electronic supplementary material). We then determine that *C*_D_ = 2.8 as a best fit to the frequency–voltage curve near hovering (*f* = 19 Hz at *V* = 5.5 V). Remarkably, this model accounts for the measurements over the entire range of voltages, as shown in figure 3*b* (*f*, dashed line). Furthermore, with the flapping frequency and thus wing speed determined the model furnishes a prediction for the lift generated, with *C*_L_ as a parameter. The lift versus voltage is shown in figure 3*b* (*L*, dashed line), where *C*_L_ = 1.2 is determined as a best fit.

With the specification of the lift and drag coefficients, the model is complete and can be used to explore variations in other parameters, for example wing size. The model bears out the reasoning that both small wings flapping quickly and large wings flapping slowly generate weak lift. In fact, our model indicates that lift is maximized at an intermediate wing size for which the flapping frequency is one-third of the zero-load speed of the motor (see electronic supplementary material). Operating near 5.5 V, for example, our motor's top speed is about 30 Hz (figure 3*a*), so this formulation recommends a target frequency of 10 Hz, to be compared with our value of 19 Hz. The recommended wing length is 11 cm, somewhat larger than our value of *R* = 8 cm. The optimal lift is predicted be 1.24 times the lift of the present prototype, an increase of a half gram that would prove useful in future efforts to support a battery.

## 7. Aerodynamic model of hovering stability

Our model can be used to assess stability by considering how the forces on the ornithopter are modified during free-flight motions. For example, as shown in figure 6*b*, a tilt induces a lift-based horizontal force, causing the body to accelerate in the direction of its lean. Once in motion, the wing speed relative to air is modified, inducing a resistive force and also a torque that depends on the centre-of-mass location. Likewise, rotations of the flyer modify the airspeed of the wings, setting up a resistive torque as well as a force. Thus, in addition to parameters that specify the wing motions, wing size *R*, total body mass *M*, and coefficients *C*_L_ and *C*_D_, free-flight stability also depends on the body moment of inertia *I* and the centre-of-mass height *h*, measured upwards from the wing tips (figure 6*a*).

These elements can be incorporated into the linearized Newton–Euler equations, thus providing a set of ordinary differential equations for body speed, tilt and tilt rate (see electronic supplementary material). The intrinsic stability of this system can then be formally assessed through an eigenvalue analysis, and in figure 6*c* we summarize how the stability properties depend on dimensionless forms of the moment of inertia and centre-of-mass height (*I/MR*^2^, *h/R*). The diagram reveals a region of stable hovering shown in grey. Experimentally, we determine *I* by supporting the (unpowered) ornithopter at a point away from the centre of mass, measuring the period of oscillations and employing the compound pendulum formula. Also, *h* is determined by hanging the ornithopter from strings attached to the body frame. These parameters reveal that our flyer (blue dot in figure 6*c*) is indeed within the stable region, and the damped tilt oscillations shown in figure 5*a* (bottom heavy) are consistent with the stable dynamics predicted by the model. Physically, this stability arises because a tilt causes horizontal motion, which then induces drag whose line of action is *above* the centre of mass and thus tends to restore the body to the upright orientation. Additionally, the torque that resists body rotations is sufficiently strong to damp oscillations.

The stability diagram also shows that high centre-of-mass body plans are unstable, with a stability boundary at *h/R* = 1/3. Indeed, our top-heavy version of the flyer lies within this region and is shown as a red dot in figure 6*c*. Furthermore, the tilt dynamics for this version (figure 5*a*, top heavy) are consistent with the divergence mode predicted by the model. In this case, the instability arises because a tilt causes horizontal motion, which then induces drag acting *below* the centre of mass, and the associated torque tends to further amplify the tilt. Interestingly, the model predicts another region of instability for sufficiently high moment of inertia and low centre of mass (lower right corner of figure 6*c*). Though difficult to investigate by modifying our current ornithopter, this prediction might be tested by using a larger body frame and thus having the motor sit even lower relative to the wings. For such an arrangement, the model predicts that the flyer would exhibit growing oscillations in tilt.

Finally, with an eye towards miniaturization, we use this model to explore how the stability characteristics depend on the size scale of the ornithopter. Interestingly, our analysis shows that the stability boundaries shown in figure 6*c* are invariant under isometric changes in scale (see electronic supplementary material). Thus, should appropriate motors enable smaller versions our model suggests that these flyers would also be stable.

## 8. Discussion

Collectively, these results illustrate a route to flapping-wing flight that involves actualizing a concept vehicle and then achieving the necessary lift, equilibrium and stability. The concept presented here is reminiscent of the swimming motions of jellyfish and involves the opening-and-closing of an aerodynamic surface. Our 10 cm prototype is designed to hover in air by drawing four wings in and out using a motor. We show that measurements of the motor torque, flapping frequency and lift can be used to inform an aerodynamic model for the forces on the wings. Given the characteristics of the motor, the model in turn predicts that fine-tuning the wing size would increase lift, which could aid in supporting an onboard battery in future versions of the flyer. Furthermore, our aerodynamic model could be modified to account for wing flexibility, which our experiments indicate is beneficial for lift production. Visualizing the unsteady flow could also inform a model that is explicitly rooted in an aerodynamic mechanism, such as vortex shedding and the generation of a downward-flowing jet owing to wing–wing interactions.

Our current ornithopter allows for the adjustment of wing motions, a capability that is critical for manoeuvring flight and for trimming the flyer to achieve hovering. Most importantly, high-speed video of free flight shows that upright stability is associated with coupled tilt and translational motions of the flyer. By expanding our model to address the changes in wing forces during such body motions, we show how the stability depends on parameters, such as the centre-of-mass location and moment of inertia. The model also predicts that scaled-down versions will exhibit stability, suggesting a promising route to miniaturization.

In the future, small-scale flapping-wing aircraft may be used in applications ranging from surveillance and reconnaissance missions to traffic and air quality monitoring. In this context and in comparison with current flapping-wing prototypes, the flyer presented here is but a step towards a feasible device. State-of-the-art ornithopters are able to achieve hovering flight by using onboard sensor feedback [10,15], external feedback [18] or additional sails [13,16,17] and tails [14] to overcome intrinsic instabilities. Our design is based on an alternative concept that exhibits intrinsic stability using flapping wings alone. Unlike the back-and-forth wing motions used in most robots, our scheme of flapping broad wings in-and-out seems to provide the strong damping of body motions needed for stability. Depending on the application, active control over an intrinsically unstable design may be more desirable than passive stability. In all cases, understanding the inherent flight dynamics is important to devising the control schemes needed for manoeuvring and for keeping upright and on course in the face of unexpected disturbances.

As schemes for locomoting through fluids, it is instructive to compare and contrast our robotic design with its biological counterparts, both flyers and swimmers. With regard to its basic kinematics, our ornithopter is most similar to a jellyfish, though our design uses four distinct wings rather than a continuous bell or umbrella. Despite this morphological difference, we expect that the inward motion of the wings generates a strong downward-flowing jet, as has been observed in flow visualization studies of swimming jellyfish [28,35] and in computational simulations [36,37]. It is also interesting to note that this jet propulsion mechanism has only been observed among aquatic organisms, such as scallops, squid and cuttlefish in addition to jellyfish [38,39]. However, similar aerodynamic mechanisms may be at work during the clap-and-fling mode of insect flight, in which the wings are brought together and peeled apart [40,41]. The general absence of jet propulsion among flying animals remains unexplained, and our realization of a hovering machine using this strategy seems to deepen this mystery.

With regard to orientational stability, this work is the first study to our knowledge that investigates the self-righting response of jellyfish-like propulsion. This is perhaps not surprising, because this mode has previously been studied only in the context of swimming in water, where the buoyancy mitigates the problems of weight support and stability [38,39]. However, jellyfish certainly contend with external flows while swimming and are able to maintain trajectory and control body orientation under such conditions [42,43]. Perhaps the stability and manoeuvrability of our ornithopter could shed light on how these animals overcome disturbances and navigate their fluid environment.
